# Vaping, Acculturation, and Social Media Use Among Mexican American College Students: Protocol for a Mixed Methods Web-Based Cohort Study

**DOI:** 10.2196/63584

**Published:** 2025-03-24

**Authors:** Bara S Bataineh, C Nathan Marti, Dhiraj Murthy, David Badillo, Sherman Chow, Alexandra Loukas, Anna V Wilkinson

**Affiliations:** 1 University of Texas Health Science Center at Houston Austin, TX United States; 2 University of Texas at Austin Austin, TX United States

**Keywords:** vaping, social media, Mexican American, college students, marketing, acculturation, protocol, artificial intelligence

## Abstract

**Background:**

The tobacco industry has a history of targeting minority communities, including Hispanic individuals, by promoting vaping through social media. This marketing increases the risk of vaping among Hispanic young adults, including college students. In Texas, college enrollment among Mexican Americans has significantly increased over recent years. However, little research exists on the link between social media and vaping and the underlying mechanisms (ie, outcome expectations, attitudes, and beliefs) explaining how vaping-related social media impacts vaping among Mexican American college students. Moreover, there is limited knowledge about how acculturation moderates the association between social media and vaping. Hispanic individuals, particularly Mexican Americans, are the largest ethnic group in Texas colleges; thus, it is crucial to understand the impact of social media and acculturation on their vaping behaviors.

**Objective:**

We outline the mixed methods used in Project Vaping, Acculturation, and Media Study (VAMoS). We present descriptive analyses of the participants enrolled in the study, highlight methodological strengths, and discuss lessons learned during the implementation of the study protocol related to recruitment and data collection and management.

**Methods:**

Project VAMoS is being conducted with Mexican American students attending 1 of 6 Texas-based colleges: University of Texas (UT) Arlington, UT Dallas, UT El Paso, UT Rio Grande Valley, UT San Antonio, and the University of Houston System. This project has 2 phases. Phase 1 included an ecological momentary assessment (EMA) study and qualitative one-on-one interviews (years 1-2), and phase 2 includes cognitive interviews and a 4-wave web-based survey study (years 2-4) with objective assessments of vaping-related social media content to which participants are exposed. Descriptive statistics summarized participants’ characteristics in the EMA and web-based survey.

**Results:**

The EMA analytic sample comprised 51 participants who were primarily female (n=37, 73%), born in the United States (n=48, 94%), of middle socioeconomic status (n=38, 75%), and aged 21 years on average (SD 1.7 years). The web-based survey cohort comprised 1492 participants self-identifying as Mexican American; Tejano, Tejana, or Tejanx; or Chicano, Chicana, or Chicanx heritage who were primarily female (n=1042, 69.8%), born in the United States (n=1366, 91.6%), of middle socioeconomic status (n=1174, 78.7%), and aged 20.1 years on average at baseline (SD 2.2 years). Of the baseline cohort, the retention rate in wave 2 was 74.7% (1114/1492).

**Conclusions:**

Project VAMoS is one of the first longitudinal mixed methods studies exploring the impact of social media and acculturation on vaping behaviors specifically targeting Mexican American college students. Its innovative approach to objectively measuring social media exposure and engagement related to vaping enhances the validity of self-reported data beyond what national surveys can achieve. The results can be used to develop evidence-based, culturally relevant interventions to prevent vaping among this rapidly growing minority population.

**International Registered Report Identifier (IRRID):**

DERR1-10.2196/63584

## Introduction

### Background

The tobacco industry has a history of targeting marketing campaigns toward specific minority groups [1,2]. In particular, Hispanic individuals, especially young adults and college students, are increasingly subjected to effective vaping promotions on social media, which raises their risk of e-cigarette use, commonly referred to as vaping [1,2]. Hispanic individuals are the largest and fastest-growing ethnic minority in the United States, comprising 19.1% of the total population, with 58.9% of this demographic being of Mexican heritage, primarily identifying as Mexican American [3]. In Texas, a majority-minority state with the second-largest Hispanic population in the United States, Hispanic individuals are the largest ethnic minority group, nearly 90% of whom are of Mexican heritage [4]. Moreover, the youthful profile of the Hispanic population, with approximately 50% aged <29 years, makes them a particularly appealing target for e-cigarette marketing [5,6]. One limitation of previous research is the study of Hispanic individuals as a homogeneous group. Despite variations in tobacco use behaviors by country of origin, there is a lack of ethnic-specific studies on Mexican Americans [7]. Moreover, little research has explored the link between social media and vaping among Hispanic college students even though national data show that they vape at rates comparable to those of their White peers and their use of social media is high [8].

Social media plays a crucial role in the acculturation process as it provides a platform for language practice, information seeking, and social interaction, all of which are integral to acculturation [9]. Social media facilitates cultural exchange by enabling users to connect, share content of cultural significance, and influence each other’s perspectives, thereby playing a pivotal role in shaping evolving cultural norms, beliefs, and identities within communities [10]. Of concern, Mexican American college students who are less acculturated yet use English-language social media might be more prone than their more acculturated peers to adopt the intrapersonal outcome expectations (eg, “e-cigarettes help me look cool and stay slim”), social norms (eg, perceived peer and family use and e-cigarettes being socially acceptable), and attitudes and beliefs (eg, e-cigarettes are safer and more convenient than cigarettes) promoted on social media, which elevates their risk of vaping [10]. Therefore, it is crucial to understand how social media influences intrapersonal mechanisms and mediators and, in turn, vaping behavior among individuals with varying levels of acculturation. This knowledge is important to developing evidence-based, culturally relevant interventions aimed at preventing vaping among this rapidly growing, vulnerable minority population.

### Objectives

The primary goals of Project Vaping, Acculturation, and Media Study (VAMoS) are to (1) identify mechanisms underlying vaping-related social media exposure and engagement and vape use among Mexican American young adults and (2) examine the role of acculturation as a moderator of these mechanisms. Project VAMoS has 3 aims, which will be implemented in 2 phases and addressed using multiple methods. Aim 1 seeks to characterize vaping-related social media and vape use from the perspective of Mexican American college students using ecological momentary assessments (EMAs) and one-on-one qualitative interviews and was completed in years 1 and 2 (phase 1). Aim 2 seeks to identify intrapersonal mediators of the relationship between exposure to vaping-related social media and subsequent vaping, and aim 3 seeks to determine the moderating role of acculturation on the direct and mediated paths of aim 2. Aims 2 and 3 are addressed using a 4-wave, biannual web-based survey in years 2 to 5 (phase 2).

**Figure 1:**
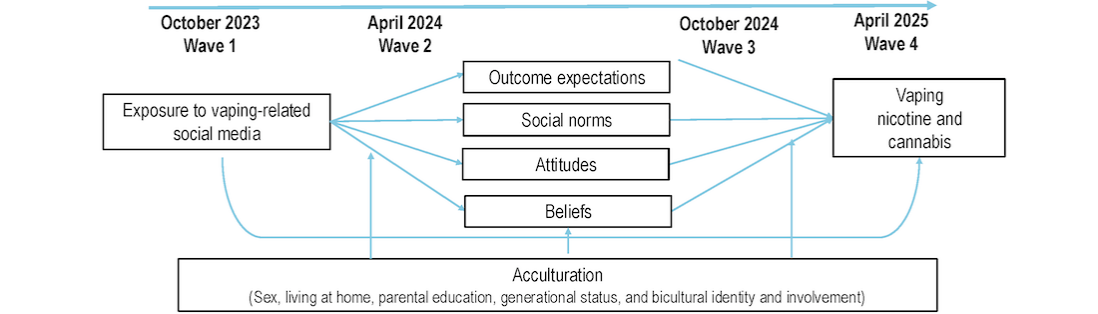
Data collection timeline and conceptual model.

Guided by social cognitive theory [11,12], we hypothesize that outcome expectations, social norms, and attitudes and beliefs will mediate the association between social media and vaping. Guided by acculturation theories [13,14], we further hypothesize that these mediated associations will be moderated by acculturation such that they will be stronger for those who use English-language media and for less acculturated students, including first-generation college students and female individuals [15,16]. Figure 1 presents the conceptual framework used to guide this study as well as the data collection timeline.

## Methods

### Overview

Project VAMoS is a 5-year study examining the independent and combined influences of social media exposure and engagement and acculturation on the vaping behaviors of a longitudinal cohort of Mexican American college students aged 18 to 29 years in Texas. VAMoS is a study that is conducted entirely on the web, including participant recruitment, data collection, and incentive delivery to participants. In this paper, we describe the protocol and present the planned mixed methodologies to be used in Project VAMoS. We present descriptive analyses of the participants enrolled in the study, focus on methodological strengths, and discuss lessons learned during the implementation of the study protocol related to recruitment and data collection and management.

### Sampling

Sampling frames were constructed by requesting names and email addresses of full- or part-time undergraduate students through Texas’s Public Information Act from the public information officers at 6 universities (University of Texas [UT] Arlington, UT Dallas, UT El Paso, UT Rio Grande Valley, UT San Antonio, and the University of Houston System). These 6 universities were selected because they are designated as Hispanic-serving institutions and all have large proportions of Hispanic students [17].

We received 100% of the requested information from all 6 colleges. Simple random samples using probability-based sampling frames of student names were drawn from each of the 6 colleges to minimize self-selection bias and improve generalizability [18]. The EMA study, qualitative interviews, and web-based surveys were and will be conducted in English, consistent with the cross-sectional web-based survey we conduct annually at Texas colleges as part of the tobacco prevention program [19]. However, participating students had the option to submit vaping-related social media content in Spanish, and our team had expertise to analyze such data.

### Study Procedure and Participants

#### Phase 1: EMA Study (Years 1-2; March 2023 to May 2023)

An introductory invitation email explaining the study’s objectives was sent to random samples of 5000 students at 5 of the 6 universities in the study (UT Arlington, UT El Paso, UT Rio Grande Valley, UT San Antonio, and the University of Houston), totaling 25,000 students, between March 24, 2023, and May 3, 2023. Eligibility criteria for the EMA study included (1) self-identifying as Mexican Americans, (2) being a degree-seeking college student, (3) being aged between 18 and 25 years, (4) being an active user of at least 2 social media platforms in the previous 30 days, (5) vaping in the previous 30 days, and (6) owning a smartphone and being willing to download the LifeData RealLife Exp mobile app.

Those who met the eligibility criteria were guided to a web-based consent form, and those who consented completed a baseline survey administered using the Qualtrics platform (Qualtrics International Inc). After completing the baseline survey, participants were invited to download the EMA app onto their smartphones via a QR code granting them access to the EMA study. Participants were provided with detailed study instructions that explained data collection procedures and offered step-by-step tutorials on how to use the app’s features to complete the daily EMAs.

In total, 2 types of EMA data were collected over the 14-day-period: daily diary and event-based assessments. Daily diaries were delivered at 10 AM each day on weekdays and at noon each day on weekends. In both cases, participants had 8 hours to complete the survey, with up to 3 reminders delivered every 2 hours. The mobile EMA system alerted participants to complete the surveys through an in-app push notification, which rang and made the phone vibrate. Event-sampling assessments were initiated by participants and were available via a button on the app home screen. Participants were instructed to initiate an event-based assessment when they (1) encountered a vaping-related post on social media or (2) vaped. For the vaping-related social media post assessments, participants had the option to upload a screenshot or photo of the post. All assessments were date and time stamped.

#### Qualitative Interviews (Years 1-2; May 2023 to November 2023)

A total of 37% (19/51) of the students who participated in the EMA also completed a 1-hour, one-on-one qualitative interview that occurred between May 2023 and November 2023. The purpose of the qualitative interviews was to more deeply understand how vaping-related social media to which participants were exposed and with which they engaged during the 14 days shaped their outcome expectations, social norms, and attitudes and beliefs (ie, mediators) and, in turn, their vaping behaviors. For example, we showed participants a set of three screenshots of vaping-related content, which was in both English and Spanish and included (1) an advertisement for various flavors of vapes, (2) a video outlining tips to quit vaping, and (3) a humorous skit about locating a friend based on the scent of their vape.

We probed their reactions to the intended message of the content; its appeal to both the interviewee and other e-cigarette users; and whether, how, and why the content enticed vaping. Findings from the qualitative interviews also provided the basis for the measures and items in the web-based survey, ensuring culturally relevant content and terminology. For example, we identified and verified the terminology used for social media concepts and vaping products and verified that the vaping products and social media platforms that we planned to assess (eg, Instagram, TikTok, and YouTube) were appropriate. If participants identified other platforms or newly emerging tobacco products, our plan was to also assess those.

The interviews were conducted in English by trained bilingual staff (to capture “Spanglish” terminology) who were either college students attending one of the participating colleges (ie, UT Rio Grande Valley) or recent graduates of the University of Texas Health Science Center (UTHealth) Houston School of Public Health. The interviews were digitally recorded and transcribed, and transcripts were analyzed using a thematic approach. Several members of our team audited the themes to ensure appropriate inferences. Each of the 19 participants was assigned a code number so that data from the EMA study and qualitative interviews could be deidentified. The code number was used to link data across the 14-day EMA study and the qualitative interviews.

#### Phase 2: Cognitive Interviews (Year 2; July 2023 to September 2023)

While designing the web-based survey, we conducted 1-and-a-half–hour cognitive interviews with 10 participants who had completed both the EMA and qualitative interviews. The purpose of the cognitive interview was to refine and finalize all measures and items for the web-based survey [20,21]. All cognitive interviews were conducted by trained staff through Microsoft Teams (Microsoft Corp) and were digitally recorded. We directed participants to complete the survey while on Microsoft Teams with both their own and project staff’s cameras off and then let us know when they finished the survey; following completion, the staff walked through each question and the participants’ answers, asking them to use the “think-aloud” method articulating their thought processes as each question was reviewed. Furthermore, the cognitive interviews incorporated specific, customized questions or “probes” regarding the survey questions. These probes yielded more detailed information than the think-aloud responses. This approach provided clarity on how participants formulated their answers as well as an estimate for approximate completion time.

#### 4-Wave Web-Based Survey Study (Years 2-5; October 2023 Until 2025)

Following the incorporation of changes and recommendations from the cognitive interviews, we began to recruit for the web-based survey. An invitation email was sent to 165,966 undergraduate students across the 6 schools describing the study and providing a link with a brief eligibility screener survey between October 2023 and December 2023. Eligibility criteria for the survey included (1) self-identifying as Mexican American, (2) being a degree-seeking college student, and (3) being aged between 18 and 29 years. Those who met the eligibility criteria were guided to a web-based consent form that included the purpose and timeline of the study and described the incentive structure. Those who consented completed a baseline survey and established the cohort for Project VAMoS.

The survey was programmed in Qualtrics and was distributed using the built-in Qualtrics Mailer. Each participant received a unique link to the survey, which could only be completed once [22]. During data collection, participants’ names, emails, and other information from the contact list were automatically saved to a secure server. Each participant was assigned a unique ID number, which allowed us to track student progress on each survey and match their data across waves without inclusion of personally identifying information in our survey database. Thus, we were able to track responses in progress and send reminders and thank-you messages to participants. In addition, this link automatically saved participants’ data as they progressed through the survey. If they needed to leave the survey before finishing, a participant could return to it at any time before the survey closed. Participants who did not complete the baseline or wave-1 survey after receiving the initial email invite received up to 4 follow-up reminders to their school email addresses.

### Measures

#### Phase 1: EMA Measures

Daily EMA measures captured data across 3 main constructs: acculturation, social media use, and vaping. For acculturation, participants provided information on their language preferences, engagement in cultural practices, and sense of ethnic identity. Regarding social media use, participants detailed how often they accessed social media per day, the time spent daily (in hours) on social media, the platforms they engaged with, the type of social media interactions they had (eg, comments, follows, likes, and shares), and their encounters with vaping-related content or advertisements. Participants also reported daily vaping behaviors, including frequency of use; device features; nicotine concentration; flavor preferences; use of other tobacco products, cannabis, and alcohol; and social contexts in which they vaped. All EMA questions were structured to capture participants’ behaviors and experiences specifically for the previous day, allowing for a comprehensive understanding of their activities over a 24-hour period. Multimedia Appendix 1 shows the constructs assessed and example measures.

#### Qualitative Interview Guides

To provide a general outline for the qualitative interviews, we drafted a qualitative interview guide consisting of questions and follow-up probes to prime participant responses. The qualitative interview guide was informed by responses obtained from the EMA study. Participants were asked about vaping and use of social media. Probes sought to determine exposure to and engagement with vaping-related social media content over the previous 14 days and how this influenced their outcome expectations, social norms, and attitudes and beliefs (ie, mediators), subsequently affecting their vaping. After conducting 10 qualitative interviews, we observed that many participants disclosed vaping not only nicotine but also cannabis or tetrahydrocannabinol (THC), with some exclusively using cannabis or THC. Therefore, we revised the guide to explore the differences that participants perceived between vaping cannabis or THC and vaping nicotine. Specifically, these questions served to tease out distinctions in use patterns, contexts, and perceptions between the 2 substances. For example, we asked questions such as the following—“Are there places where and/or times when you use nicotine versus THC?”—to better understand the situational and behavioral differences in vaping these products.

#### Phase 2: 4-Wave Web-Based Survey Study

At wave 1 (baseline), participants completed a self-report survey consisting of 142 measures. Measurement selection was guided by the conceptual model in Figure 1, informed by the EMA and qualitative interviews, and refined through the cognitive interviews. Vaping measures were selected or adapted from national surveys, including the Population Assessment of Tobacco and Health Study, the Behavioral Risk Factor Surveillance System, the Youth Tobacco Survey, and the National Survey on Drug Use and Health [23-26] (Multimedia Appendix 1).

The constructs assessed included sociodemographic characteristics, susceptibility to vaping, patterns of nicotine and cannabis or THC use, symptoms of dependence, cessation, outcome expectations from vaping, prevailing social norms, attitudes and beliefs, exposure to and engagement with social media, social media use patterns, and potential problems with social media [27]. We also assessed acculturation using proxy measures such as country of birth [28] and scaled measures such as the American Identity Measure [29-31] and included correlates of quality of family interactions and relationships [32]. Covariates assessed confounders and included use of other tobacco products and alcohol, experiences of racial or ethnic discrimination [33], and symptoms of depression [34] and anxiety [35].

At wave 2, we made several revisions. First, we shortened the survey by removing all items that would not change over time, such as sex at birth. We also did not ask participants to respond to “ever use” items at wave 2 (eg, ever used e-cigarettes) if they responded affirmatively at wave 1. Second, we included a limited number of new items; for example, we collected participants’ race as defined by the US Census Bureau and intrapersonal measures of impulsivity, sensation seeking, and social anxiety [36-39]. Third, we asked participants to provide their personal social media handles or usernames for YouTube, Instagram, X (formerly known as Twitter), and Reddit to objectively assess the vaping-related social media content to which participants were exposed. We also asked participants to share 3 subreddits to which they subscribed and with which they frequently engaged also for objective assessment of vaping-related social media content. Consistent with our previous research [40,41], these data will be collected via the YouTube application programming interface (API) and web scraping for Instagram and X [42]. We will search for publicly available posts, videos, and hashtags with which our participants engage. We will not have access to the posts that participants see in their feeds or “for you” pages as these are not publicly available data and, thus, are outside the scope of our institutional review board (IRB) protocol.

Before analysis, all personally identifying information will be replaced with a unique ID number that matches the participants’ ID number for the web-based survey, allowing us to link all the data for analysis. Custom-developed Python scripts (Python Software Foundation) will be used for API data collection from YouTube, and the Selenium toolkit [43] or Zeeschuimer [44] will be used for all browser-based web scraping (Instagram, YouTube, and X). The Python Reddit API Wrapper [45] will be used to collect data from Reddit. Depending on the platform, the date range of available posts will vary. We will scrape as much publicly available data as possible. The collected social media data will be studied computationally (with manual content analysis used to check validity). In terms of the former, we will use topic modeling, a computational natural language processing technique that we have successfully used in previous work [46], to identify statistically significant themes from (1) the posts of each user, (2) the aggregated posts of all users, and (3) the posts from subreddits that users follow and that they provided to us. Particularly for the latter, the data will be voluminous. Previous work has established the utility of generative artificial intelligence as a topic modeling tool to discover latent themes in large volumes of unstructured textual data [47]. We will adapt prompts developed and tested by others for topic modeling using ChatGPT (OpenAI) [48] to provide up to 5 themes for each user, 15 themes for all aggregated users, and 15 themes for each subreddit. We will then conduct a manual content analysis of a random sample of 150 posts from the aggregated posts of all users and the posts from subreddits that users follow to evaluate the robustness of the ChatGPT-based results. Our use of generative artificial intelligence will provide objective assessments of vaping-related social media content, which will allow us to characterize content that appeals to and entices Mexican American college students to use vapes**.**

### Ethical Considerations

#### Human Participant Ethics Review Approvals

The IRB at UTHealth Houston (HSC-SPH-21-0976) approved all aspects of the study protocol. UT Austin approved the reliance on the UTHealth Houston IRB.

#### Informed Consent

All eligible participants in this study provided electronic informed consent before their participation in the 2 phases. Participants were informed about the study’s objectives, procedures, potential risks, and benefits. They were informed that their participation was voluntary and that they could withdraw from the study at any time.

#### Privacy and Confidentiality

All collected data were deidentified to protect participants’ privacy at the baseline wave. Personal information is removed before analysis, and the data are securely stored, with access restricted to authorized personnel.

#### Compensation Details

##### Phase 1

Participants who completed the EMA study were compensated with e-gift cards (US $10-$50) based on the percentage of daily diary assessments completed over the study period, with a minimum requirement of 50% of assessments completed to receive an incentive. Those who completed at least 7 daily diaries over the 14-day period received US $10 in e-gift cards (5/51, 10% of the participants), those who completed at least 10 daily diaries received US $35 in e-gift cards (13/51, 25%), and those who completed ≥13 of their daily diary EMAs received US $50 in e-gift cards (28/51, 55%). Participants were not compensated for event-based EMAs so as not to encourage false reporting of vaping or encounters with vaping-related social media content. Participants who completed the qualitative interviews were compensated with a US $50 e-gift card. Individual incentives were distributed within 2 weeks of completing the EMA and qualitative interviews.

##### Phase 2

**Figure 2:**
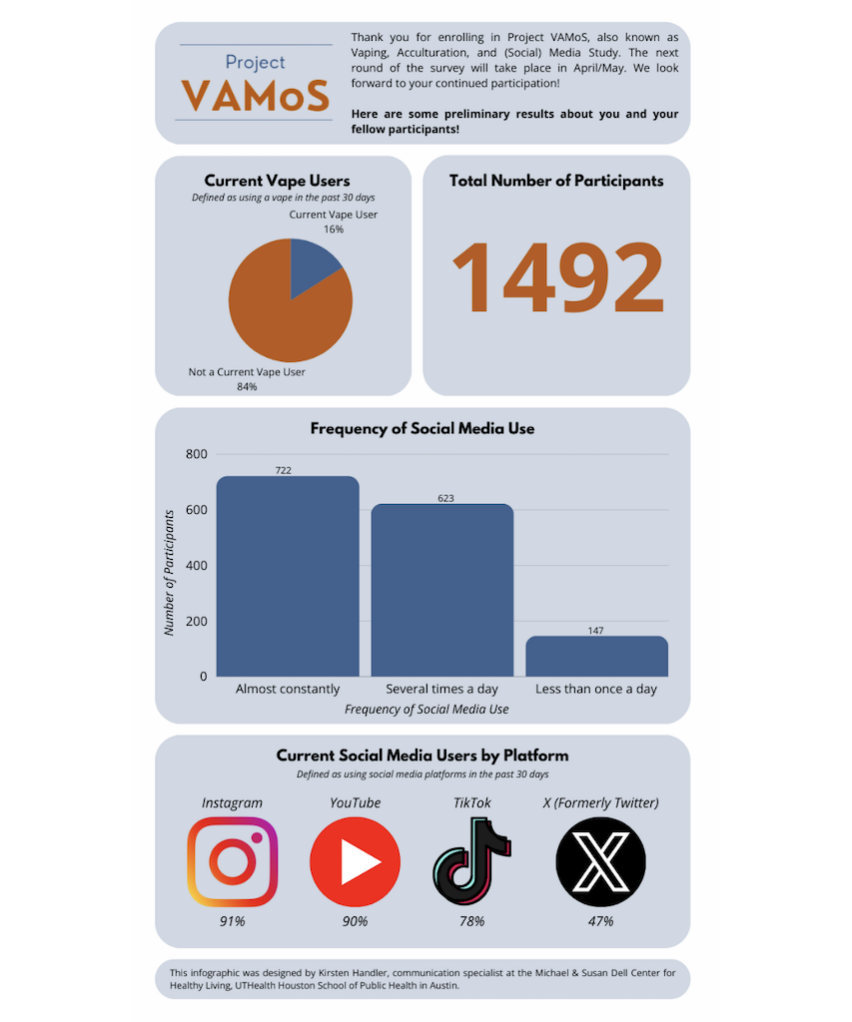
Infographic distributed between baseline and wave 2 of the web-based survey.

Participants who completed the cognitive interviews were compensated with a US $50 e-gift card, which was distributed within 2 weeks of completing the interview. Individual incentives were and will be offered at each of the 5 waves of the web-based survey. Students receive a US $25 e-gift card for completing each of the first 4 waves (totaling US $100) and a larger e-gift card of US $30 for completing the fifth and final wave. In addition, to maximize retention, we send an infographic with preliminary results between each survey (see Figure 2 for the infographic distributed between waves 1 and 2). Individual incentives are distributed within 2 weeks of survey completion.

### Data Integrity and Security

We used rigorous protocols and advanced security measures to protect the integrity of the web-based survey data against bots and fraudulent respondents. First, we created a contact list with email addresses provided by each college and then distributed the survey using a unique, 1-time-use link for each participant in Qualtrics [22]. This setting prevented multiple survey completion attempts from the same individual by placing a cookie on the participant’s browser when they submitted a response. Participants are only able to complete the survey once; if the respondent clicks on the survey link after completing it, the presence of the cookie will prevent them from beginning a new survey. In addition, we incorporated a Completely Automated Public Turing Test to Tell Computers and Humans Apart (CAPTCHA) to ensure that surveys are completed by humans, not automated bots. Other strategies used to maximize the validity of the web-based survey data include the use of skip patterns within and across waves, which minimize participant fatigue and eliminate inconsistent responses; the use of “soft prompts” in most survey pages so that participants who skip questions are asked to verify their intent to leave the question blank; and the display of a limited number of items per screen or page [49,50]. In addition, participants are given the option to review and modify their responses using a Back button to enhance data accuracy. At wave 2, we added a human and attention check item to further ensure data validity and reliability [49].

Data security in Qualtrics is supported through advanced technological measures and robust policy enforcement. Qualtrics uses transport layer security encryption (also known as HTTPS) for all transmitted data. Qualtrics services are hosted by trusted data centers that are independently audited using the industry standard Statement on Standards for Attestation Engagements 18 method [49]. Access to datasets containing personal or identifying information is strictly limited to authorized members of the research team. These individuals undergo training in data privacy, confidentiality, and security protocols. Furthermore, they must use secure, complex passwords to access the Qualtrics data servers.

### Final Data Quality Checks

Additional data quality checks were and will continue to be implemented to ensure the highest-quality survey data. Our team thoroughly reviews the survey responses for evidence of poor data quality. For example, we search for discrepancies in responses, such as participants providing contradictory answers to questions that should logically correlate or displaying a potentially problematic pattern of responses (eg, straight lining or selecting the same answer option several times in a row and a completion time of <10 minutes). Despite the exclusion of 0.2% (4/1824) of the participants at wave 1 due to these quality concerns, these participants received their incentive for taking part in this phase of the survey after we verified that a proper email address had been provided.

### Maximizing Retention

To optimize retention across all waves of data collection, our team (1) fosters participant identification with the study by using our project name on all materials and correspondence [50-52]; (2) makes participation convenient by allowing participants to respond to the survey on a computer, smartphone, or other personal devices anywhere and at any time during the survey period [50]; (3) shares key study results via an infographic every 6 months; (4) sends multiple reminders (starting at wave 2, we sent reminders via email and SMS text message); and (5) keeps the survey open for up to 6 weeks to accommodate students’ school and work schedules.

## Results

### Overview

Funding for Project VAMoS began in August 2022 and will conclude in March 2027. Data collection for phase 1 (EMA and qualitative interviews) was completed by June 2023. Phase 2, wave 1, was completed between November 2023 and December 2023; wave 2 was completed between April 2024 and May 2024. Wave 3 was completed between October 2024 and November 2024, and wave 4 will be completed between April 2025 and May 2025 (Table 1).

**Table 1:** Vaping, Acculturation, and Media Study timeline. Timeline: August 1, 2022-March 31, 2027.

		Year 1 (2022-2023)		Year 2 (2023-2024)		Year 3 (2024-2025)		Year 4 (2025-2026)		Year 5 (2026-2027)	
		August-January	February-July	August-January (wave 1)	February-July (wave 2)	August-January (wave 3)	February-July (wave 4)	August-January	February-July	August-January	February-March
**Phase 1: survey development and qualitative interviews**											
	Survey development	✓	✓								
	Ecological momentary assessment		✓								
	Qualitative interviews		✓	✓							
**Phase 2: cognitive interviews and data collection**											
	Cognitive interviews		✓	✓							
	Survey refinement		✓	✓							
	Recruitment of participants			✓							
	Survey data collection			✓	✓	✓	✓				
	Data collection from social media				✓	✓	✓	✓			
	GenAI^a^ classification					✓	✓	✓	✓	✓	
	Participant retention				✓	✓	✓				
	Cleaning and analyzing data		✓	✓	✓	✓	✓	✓	✓	✓	✓

^a^GenAI: generative artificial intelligence.

### EMA Eligibility and Completion Rates

**Figure 3:**
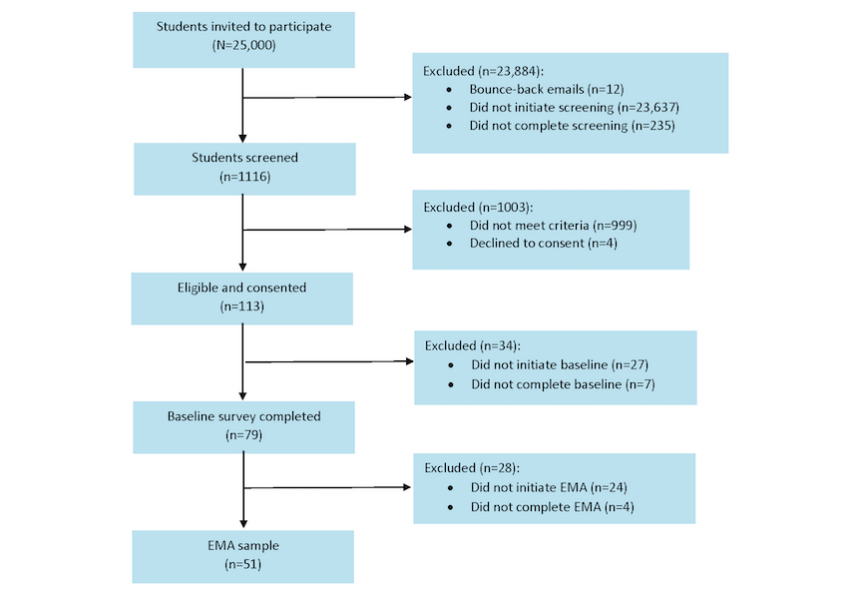
Flow diagram of ecological momentary assessment (EMA) participant recruitment.

In spring 2023, a total of 25,000 invitations were sent to potential participants, and 1116 (4.5%) students were screened to participate in the EMA study, of whom 113 (10.1%) were eligible and 79 (7.1%) completed a baseline survey that collected information on their demographics, social media use, and tobacco use behaviors. Of the 79 eligible participants, 24 (30%) did not proceed with the EMA study, and 4 (5%) were dropped due to inactivity (ie, did not complete the daily assessments). Thus, 45.1% (51/113) of the participants took part in the 14-day EMA study to address aim 1 (Figure 3). Completion rates were calculated to determine the feasibility of EMA as a means of obtaining daily data among Mexican American college students. Of a possible 714 prompts, a total of 608 (85.2%) were sent to participants over 14 days; this discrepancy is due to some participants disabling the LifeData app before the study concluded, meaning that they were not able to receive further prompts. Of the 608 prompts sent, we received 583 (95.9%) complete responses. Of the overall sample of 51 participants, 5 (10%) completed at least 10 daily diaries, 13 (25%) completed at least 11, and 28 (55%) completed ≥13 of the daily diary EMAs. The average completion rate was 83.2% (SD 24.9%; range 7%-100%).

### 4-Wave Web-Based Survey Eligibility and Retention Rates

**Figure 4:**
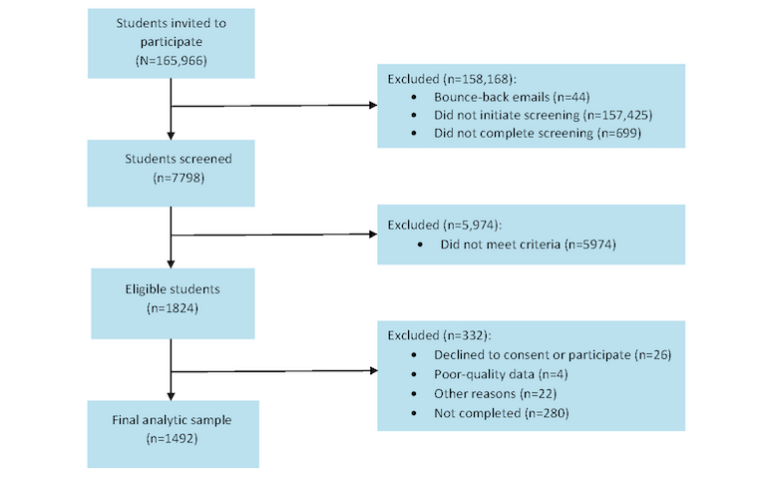
Flow diagram of web-based survey participant recruitment.

In fall 2023, a total of 165,966 invitations were sent to potential participants, and 7798 (4.7%) students were screened to participate, of whom 1824 (23.4%) were eligible (Figure 4). Of those 1824 eligible participants, 26 (1.4%) declined to consent, 4 (0.2%) were excluded due to poor-quality data, 22 (1.2%) did not meet the age criteria and were excluded, and 280 (15.4%) did not complete the survey. Thus, 81.8% (1492/1824) of Mexican American students were recruited from all 6 participating universities to establish our cohort and address aims 2 and 3 (Figure 4). Of the baseline cohort, the retention rate in wave 2 was 74.7% (1114/1492).

### Sample Characteristics

For the purposes of this paper, descriptive statistics were used to summarize the characteristics of the EMA study participants (Table 2) and the baseline characteristics of the participants who form the cohort for the web-based survey (Table 3). The final EMA analytic sample comprised 51 current vaping and social media users who were of Mexican American heritage. Participants (Table 3) were primarily female (37/51, 73%), born in the United States (48/51, 94%), of a middle socioeconomic status (38/51, 75%), and aged 21 years on average (SD 1.7 years). The final sample for the web-based survey included 1492 participants who were of Mexican American; Tejano, Tejana, or Tejanx; or Chicano, Chicana, or Chicanx heritage. Participants (Table 3) were primarily female (1042/1492, 69.8%), born in the United States (1366/1492, 91.6%), of a middle socioeconomic status (1174/1492, 78.7%), and aged 20.1 years on average (SD 2.2 y).

**Table 2:** Descriptive statistics for the ecological momentary assessment sample (N=51).

Characteristic		Values
Age (y), mean (SD)		21 (1.7)
**Sex, n (%)**		
	Male	14 (27)
	Female	37 (73)
**Country of birth, n (%)**		
	United States	48 (94)
	Mexico	3 (6)
**Socioeconomicstatus**, **n (%)**		
	Low (1-3)	4 (8)
	Middle (4-7)	38 (75)
	High (8-10)	9 (18)
Socioeconomic status, mean (SD)		5.9 (1.7)
**Federal Pell Grant, n (%)**		
	Yes	40 (78)
	No	11 (22)
**School attended,** **n (%)**		
	UT^a^ Arlington	11 (22)
	UT El Paso	12 (24)
	UT San Antonio	10 (20)
	UT Rio Grande Valley	10 (20)
	University of Houston System	8 (16)

^a^UT: University of Texas.

**Table 3:** Descriptive statistics for the survey sample at wave 1 (N=1492).

Characteristic			Values
Age (y), mean (SD)			20.1 (2.2)
**Sex, n (%)**			
	Male	450 (30.2)	
	Female	1042 (69.8)	
**Country of birth, n (%)**			
	United States	1366 (91.6)	
	Mexico	114 (7.6)	
	Other	12 (0.8)	
**Socioeconomicstatus**, **n (%)**			
	Low (1-3)	260 (17.4)	
	Middle (4-7)	1174 (78.7)	
	High (8-10)	56 (3.8)	
Socioeconomic status, mean (SD)			4.9 (1.5)
**Federal Pell Grant, n (%)**			
	Yes	474 (31.8)	
	No	1018 (68.2)	
**School attended,** **n (%)**			
	UT^a^ Arlington	264 (17.7)	
	UT El Paso	240 (16.1)	
	UT San Antonio	405 (27.1)	
	UT Rio Grande Valley	321 (21.5)	
	University of Houston System	195 (13.1)	
**Year in college, n (%)**			
	Freshman	522 (35)	
	Sophomore	368 (24.7)	
	Junior	295 (19.8)	
	Senior	307 (20.6)	

^a^UT: University of Texas.

## Discussion

### Expected Findings

Project VAMoS is one of the first longitudinal mixed methods research projects to explore the influences of social media and acculturation on vaping behaviors focusing exclusively on Mexican American college students. The results of this project will provide evidence for the roles of intrapersonal mediators (eg, outcome expectations and social norms) in the associations between vaping-related social media content and subsequent vaping. The results will also determine the moderating role of acculturation in the association between vaping-related social media content and subsequent vaping, as well as in the mediated paths between vaping-related social media content and subsequent vaping. In turn, results can inform the tailoring of culturally relevant interventions and the development of countermarketing policies to prevent vaping among Mexican American college students [53].

Project VAMoS is innovative in 3 key areas. First, unlike many studies that consider Hispanic individuals as a homogeneous group, this project specifically focuses on Mexican Americans, acknowledging the diversity within this population, particularly in tobacco use patterns related to country of origin and acculturation levels [54]. Second, the project delves into the underlying mechanisms or mediators—such as outcome expectations, social norms, and attitudes and beliefs—that influence how exposure to vaping-related social media content affects vaping behavior. These mediators are identified through an EMA study and one-on-one qualitative interviews and are empirically examined in a comprehensive, 4-wave survey study. Finally, in addition to subjective assessments, the project conducts objective assessments of the social media content that participants actually view and engage with on popular platforms such as Instagram, TikTok, and YouTube. This approach minimizes the biases often associated with self-reported data [55].

### Refining and Finalizing the Web-Based Survey

Some items were developed or refined based on findings from the EMA study and one-on-one interviews and then tested through cognitive interviewing to ensure their appropriateness for the study population [11,12]. For example, given the diverse range of substances that can be vaped, we incorporated options such as cannabis, THC, or delta-9; delta-8 THC; cannabidiol; caffeine; essential oils; vitamins; and more to include more potential vaping choices. In addition, we refined survey questions to ensure that they specifically addressed the substances that participants are vaping by including the terms “with nicotine” or “with cannabis/THC” without assuming exclusive consumption of either substance. As another example, we added response options such as “I do not know” or “neutral,” along with time frames such as “When you were younger?” or “currently” to some acculturation measures. Moreover, we amended and will continue to amend items that are and are not included in subsequent waves in response to findings from the baseline survey wave. We will also add items in response to changes in the marketplace, such as the introduction of a new vaping device type, or in the policy landscape, such as a state or federal ruling regarding vapes or the marketing and promotion of these products.

Items were also added in response to the 2023 Surgeon General Report titled Social Media and Youth Mental Health [56], which concluded that there are gaps in current knowledge related to “the mental health impacts posed by social media” and that, currently, we cannot conclude that social media is sufficiently safe for young people. Accordingly, at wave 1, we assessed symptoms of depression, suicidal ideation, and anxiety [34,35]. At wave 2, we added measures to assess sensation seeking and impulsivity [38,39]. Symptoms of depression and anxiety increase risk of vaping both nicotine and cannabis or THC [57,58], whereas sensation seeking is associated with the uptake of vaping nicotine [59,60] and impulsivity increases risk of current vaping [61]. Therefore, the inclusion of these mental health and personality constructs complements existing measures assessed in Project VAMoS and will enable us to examine their role as potential mediators and moderators of the association between social media use and vaping.

### Lessons Learned

Recruiting participants to join the EMA took more time than planned. Thus, we revised the eligibility criteria from vape use in the previous week to use in the previous 30 days. Similarly, recruiting participants to join the web-based survey took longer than planned; thus, we expanded the age range eligibility criteria from 18 to 25 years to 18 to 29 years and expanded recruitment from freshman students to all undergraduate levels, and we sent up to 4 email reminders rather than just 3. However, recruitment for the EMA study was completed in 6 weeks, and recruitment for the web-based survey was completed in 8 weeks. These adjustments broadened our participant pool, thereby creating a more representative cohort of college students. These challenges might be due to the specific nature of the inclusion criteria, such as being Mexican American [62]; potential hesitancy among young adults to participate in substance use research studies [62-65]; or the omission of incentive details from the invitation emails sent to participants to comply with IRB requirements.

This project provides an opportunity to better understand how Mexican American college students self-identify across racial categories. In wave 1, a total of 78.2% (1167/1492) of the participants identified solely as Hispanic when responding to a combined race and ethnicity question, whereas only 20.2% (301/1492) specified their race (data not shown). In response, at wave 2, we adopted a best practice approach aligning with the US Census Bureau’s guidelines [66,67]. Specifically, we separated the race and ethnicity question into 2 items on the survey to allow participants to identify with a specific race while also recognizing their Hispanic ethnicity. Such an approach ensures that our data more accurately reflect the diverse racial backgrounds within the Mexican American population, enhancing our understanding of the associations among race, ethnicity, and social factors in the Mexican American community [66,67].

### Strengths and Limitations

This project has many strengths. A major strength is the focus on Mexican American college students. Most studies have examined Hispanic populations as a single group, overlooking the differences in vape use patterns influenced by country of origin and acculturation levels. In addition, the Mexican American population in Texas is on the rise, with an increasing number of young individuals from this group enrolling in college annually [68]. Another major strength is the innovative approach in objectively measuring both exposure to and engagement with social media related to vaping. These objective assessments optimize the validity of self-reported social media exposure and engagement data and represent a distinct advantage that cannot be replicated by national surveys such as the Population Assessment of Tobacco and Health Study [23]. Furthermore, our EMA study and in-depth semistructured interviews enhance our research by enabling us to uncover culturally pertinent terms and concepts for evaluation in our web-based survey. Finally, the longitudinal design of our web-based survey allows for mediation tests to identify the intrapersonal factors that explain whether and how baseline levels of vaping-related social media content impact subsequent vape use at follow-up [69].

The project is not without limitations. First, we focused on college students, which limits generalizability. Nonetheless, this drawback is somewhat mitigated by the rapidly growing rate of college enrollment among Mexican American young adults. Second, we only recruited participants from Texas. However, Texas is a majorly minority state with the second-largest Hispanic population in the United States, predominantly consisting of Mexican Americans [4]. Moreover, Texas has the second-largest population of Hispanic college students in the United States [68]. This demographic profile enables us to concentrate on the largest Hispanic subgroup in the country.

### Implications

The results of this research will offer unique insights into the prevalence, patterns of use, and determinants contributing to vaping among Mexican American college students. This information will inform the development of evidence-based, culturally relevant prevention interventions and policies specifically for the growing population of Mexican American college students. Moreover, results from mediation analyses will pinpoint the most effective intervention targets (namely, significant mediators), and the outcomes of such analyses will highlight which subgroups (namely, significant moderators) are most at risk from the impacts of exposure to and engagement with vaping-related social media content.

The results gathered from the EMA study, in-depth semistructured interviews, and objective assessment of social media content will reveal the vaping messaging that attracts Mexican American college students to vaping and influences them to vape. These insights will then serve as a foundation for developing economical counteradvertising and health messaging strategies. These strategies can be effectively disseminated through social media platforms to reduce vape use among Mexican American college students, thereby lowering their risk of tobacco-related morbidity and mortality. Finally, research in this area contributes to the broader goal of health equity by ensuring that the health needs of ethnic minority groups are adequately addressed. By understanding and addressing the specific health behaviors and their determinants within minority communities, public health initiatives can move toward reducing health disparities.

## Appendix

Multimedia Appendix 1 Examples of ecological momentary assessment and web-based survey measures.

## Abbreviations

API: application programming interface
EMA: ecological momentary assessment
IRB: institutional review board
THC: tetrahydrocannabinol
UT: University of Texas
UTHealth: University of Texas Health Science Center
VAMoS: Vaping, Acculturation, and Media Study

## Data Availability

The datasets generated or analyzed during this study are not publicly available as the study is ongoing but are available from the corresponding author on reasonable request.
