# Correction: Vaping, Acculturation, and Social Media Use Among Mexican American College Students: Protocol for a Mixed Methods Web-Based Cohort Study

**DOI:** 10.2196/95651

**Published:** 2026-04-02

**Authors:** Bara S Bataineh, C Nathan Marti, Dhiraj Murthy, David Badillo, Sherman Chow, Alexandra Loukas, Anna V Wilkinson

**Affiliations:** 1 University of Texas Health Science Center at Houston Austin, TX United States; 2 University of Texas at Austin Austin, TX United States

In “Vaping, Acculturation, and Social Media Use Among Mexican American College Students: Protocol for a Mixed Methods Web-Based Cohort Study” [1], an error in the institutional review board number was identified in the Methods section under the subheading Human Participant Ethics Review Approvals.

The following statement has been corrected:

The IRB at UTHealth Houston (HSC-SPH-19-0796) approved all aspects of the study protocol.

The statement now reads:

The IRB at UTHealth Houston (HSC-SPH-21-0976) approved all aspects of the study protocol.

The correction will appear in the online version of the paper on the JMIR Publications website, together with the publication of this correction notice. Because this was made after submission to PubMed, PubMed Central, and other full-text repositories, the corrected article has also been resubmitted to those repositories.
