# Supplementary material for: Vaping, Acculturation, and Social Media Use Among Mexican American College Students: Protocol for a Mixed Methods Web-Based Cohort Study
Source: JMIR Res Protoc. 2025 Mar 24;14:e63584. doi: 10.2196/63584 (PMC11976173; doi:10.2196/63584)
Supplement: Multimedia Appendix 1 [file resprot_v14i1e63584_app1.docx]

**Multimedia Appendix 1**. Examples of ecological momentary assessment (EMA) and web-based survey measures.

| Construct | | EMA questions | Web-based survey questions |
| --- | --- | --- | --- |
| **Exposure** | | | |
|  | Social media use | - “Yesterday, did you use social media?” Response options: “No” and “Yes” | - “In the past 30 days, which of the following social media platforms did you use? Please select all that apply.” Response options (multiple selections allowed): “Instagram,” “TikTok,” “YouTube,” and “Twitter/X” |
|  | Vaping-related social media | - “Yesterday, when you saw e-cigarette ads or content on social media platforms from where did it originate?” Response options: “E-cigarette industry-sponsored account,” “An influencer/celebrity account,” “Other social media user account,” and “Don’t know” | - “Have you ever seen, liked or posted/re-posted e-cigarette content on any social media platforms, even one time?” Response options: “Yes” and “No” |
| **Mediators** | | | |
|  | Outcome expectations | - Not assessed | - “How much do you agree or disagree with the following statements even if you don’t use e-cigarettes? I think using e-cigarettes to vape nicotine...   - ...could help me stay slim.   - ...could help when I’m feeling stressed.   - ...could help relax me.   - ...could help when I’m feeling anxious.   - ...could help energize me.   - ...could help me concentrate.   - ...could give me a definite nicotine hit.   - ...could help me have fun.   - ...could help me have more friends.   - ...could help me look cool or fit in with peers.   - ...could help feel older or more mature.   - ...could help me fit in with my family.” - Response options: “Strongly disagree,” “Disagree,” “Neither agree nor disagree,” “Agree,” and “Strongly agree” |
|  | Social norms | - Not assessed | - “How much do you think your closest friends approve of using e-cigarettes?” Response options: “Strongly disapprove,” “Disapprove,” “Somewhat disapprove,” “Neither disapprove nor approve,” “Somewhat approve,” “Approve,” and “Strongly approve” |
|  | Attitudes and beliefs | - Not assessed | - “How addictive are...   - ...e-cigarettes with nicotine?   - ...cigarettes?” - Response options: “Not at all addictive,” “Somewhat addictive,” and “Very addictive” |
| **Moderators** | | | |
|  | Acculturation—proxy | - “Yesterday, what languages did you speak at home and at work/school?” Response options: “English,” “Spanish,” and “Both English and Spanish” | - “In which country was your father born?” Response options: “Mexico,” “United States,” “Other (fill in): [text entry field],” and “I don’t know/not applicable” |
|  | Acculturation—direct | - “Yesterday, I had a strong sense of belonging to my own ethnic group.” Response options: “Strongly disagree,” “Disagree,” “Neither agree nor disagree,” “Agree,” and “Strongly agree | - “Tell me how much you agree with the following:   - I find it easy to harmonize Mexican and American cultures.   - I rarely feel conflicted about being bicultural.   - I find it easy to balance both Mexican and American cultures.   - I do not feel trapped between Mexican and American cultures.   - I cannot ignore the Mexican or American side of me.   - I feel Mexican and American at the same time.   - I relate better to a combined Mexican-American culture than to Mexican or American culture alone.   - I feel Mexican-American.   - I feel part of a combined culture.” - Response options: “Strongly disagree,” “Disagree,” “Neither agree nor disagree,” “Agree,” and “Strongly agree” |
| **Vaping outcomes** | | | |
|  | Vape use | - “Yesterday, did you use an e-cigarette?” Response options: “Yes” and “No” | - “Have you ever used an e-cigarette to vape nicotine, even one or two puffs?” Response options: “Yes” and “No” |
|  | Susceptibility | - Not assessed | - “Do you think you will use an e-cigarette to vape nicotine in the next 12 months?” Response options: “Definitely not,” “Probably not,” “Probably yes,” and “Definitely yes” |
|  | Dependence | - Not assessed | - “How soon after you wake up do you typically use your first e-cigarette to vape nicotine?” Response options: “Within 5 minutes of waking,” “6-30 minutes after waking,” “31-60 minutes after waking,” and “I am not a daily user of e-cigarettes with nicotine” |
| **Covariates** | | | |
|  | Other tobacco use | - “Yesterday, did you use any of the following?   - Cigarettes   - Cigars, cigarillos, little cigars   - Hookah   - Smokeless tobacco   - Other types of nicotine (e.g., nicotine patches, gum/lozenges, gummies, etc.)” - Response options: “Yes” and “No” | - “On how many of the past 30 days did you smoke a cigarette?” Response options: “0,” “1,” “2”... “30” |
|  | Alcohol or cannabis or THC^a^ use | - “Yesterday, did you use any of the following?   - Vaped marijuana   - Smoked marijuana   - Edible marijuana” - Response options: “Yes” and “No” | - “During the past 30 days, on how many days did you have at least one drink of alcohol?” Response options: “0 days,” “1 or 2 days,” “3 to 5 days,” “6 to 9 days,” “10 to 19 days,” “20 to 29 days,” and “All 30 days” |
|  | Physical health | - Not assessed | - “During the past 14 days, how much have you been bothered by any of the following problems?   - Stomach or bowel problems   - Back pain   - Pain in your arms, legs, or joints   - Headache   - Chest pain or shortness of breath   - Dizziness   - Coughing   - Wheezing” - Response options: “Not at all,” “A little bit,” “Somewhat,” “Quite a bit,” and “Very much” |
|  | Mental health | - “Thinking about how you felt yesterday, mark the response that most applies.   - I felt anxious.   - I felt happy.   - I felt calm.” - Response options: “Strongly disagree,” “Disagree,” “Neither agree nor disagree,” “Agree,” and “Strongly agree” | - “Over the last 2 weeks, how often have you been bothered by the following problems?   - Little interest or pleasure in doing things   - Feeling down, depressed, or hopeless   - Trouble falling or staying asleep, or sleeping too much   - Feeling tired or having little energy   - Poor appetite or overeating   - Feeling bad about yourself or that you are a failure and have let yourself or your family down   - Trouble concentrating on things, such as reading the newspaper or watching TV   - Moving or speaking so slowly that other people have noticed, or the opposite, being so fidgety or restless that you have been moving around a lot more than usual   - Thoughts that you would be better off dead or of hurting yourself in some way” - Response options: “Not at all,” “Several days,” “More than half the days,” and “Nearly every day” |

^a^THC: tetrahydrocannabinol.
